# Morbihan syndrome: a case report and literature review*

**DOI:** 10.1590/abd1806-4841.20164291

**Published:** 2016

**Authors:** Rossana Cantanhede Farias de Vasconcelos, Natália Trefiglio Eid, Renata Trefiglio Eid, Fabíolla Sih Moriya, Bruna Backsmann Braga, Alexandre Ozores Michalany

**Affiliations:** 1 Universidade de Santo Amaro (Unisa) – São Paulo (SP), Brazil; 2 Faculdade de medicina de São José do Rio Preto (Famerp) – São Paulo (SP), Brazil

**Keywords:** Edema, Erythema, Rosacea

## Abstract

Morbihan syndrome is a rare entity that more commonly affects women in the third or fourth decade of life. It is considered a special form of rosacea and its pathogenesis is not fully known. It is clinically characterized by the slow appearance of erythema and solid edemas on the upper portion of the face, with accentuation in the periorbital region, forehead, glabella, nose, and cheeks. We report the case of a patient presented with edema on the upper eyelid for a year. These findings suggested the diagnosis of Morbihan syndrome. We aim to report a rare, particularly refractory and chronic form of rosacea, which has received little attention in the literature.

## INTRODUCTION

Morbihan disease was first reported in 1957 by Robert Degos.^1^ It is believed that “Morbihan syndrome” is a more correct term in consideration of different etiopathogenic factors.

Morbihan syndrome is a rare entity that mostly affects Caucasian adults of both sexes. Only one black and one Indian male patient were reported.^2,3^ The pathogenesis of the syndrome is not well elucidated.^4^ According to most authors, it is a clinical variety of acne or rosacea, a common episodic chronic cutaneous disorder that affects the face. It is characterized by the permanent presence of erythema accompanied by telangiectasia, with frequent mixed facial flushing, papules, pustules, diffuse edema, and nodules.^5,6^

According to some authors, Morbihan syndrome can be caused by abnormalities in lymphatic vessels.^7^

Clinically, the syndrome is characterized by the slow appearance of erythema and solid edemas on the upper portion of the face, with accentuation in the periorbital region, forehead, glabella, nose, and cheeks.^8^ The cutaneous lesions persist indefinitely with no tendency to spontaneous involution without treatment. Lesions are initially floating and then permanent, causing swelling and distortion of facial contours.^8^ As the persistent facial edema can lead to visual impairment in severe cases, control of the disease activity is essential.

Laboratory results are nonspecific or not found, histopathology and staining should be performed to rule out other conditions.

Differential diagnoses include orofacial granulomatosis, sarcoidosis, Hansen’s disease, systemic lupus erythematosus, cutaneous leishmaniasis, foreign body granuloma, facial granuloma, superior vena cava syndrome, and scleredema of Buschke.^4^ Moreover, barbiturates, chlorpromazine, diltiazem, and isotretinoin can induce clinical manifestations similar to Morbihan syndrome.

A number of treatment options are suggested with several systemic drugs used in high doses for a prolonged period. However, not all patients respond to treatment.

The aim of this study was to report a Morbihan disease patient with refractory and chronic rosacea, a rare case that has received little attention in the literature.

## CASE REPORT

We report a 39-year-old male patient complaining of swelling of the upper eyelids for a year. He denied pain and itching and reported worsening of the edema after sun exposure. He also denied any other comorbidity and medication use. He reported worsening of symptoms in the last week.

**Figure 1:**
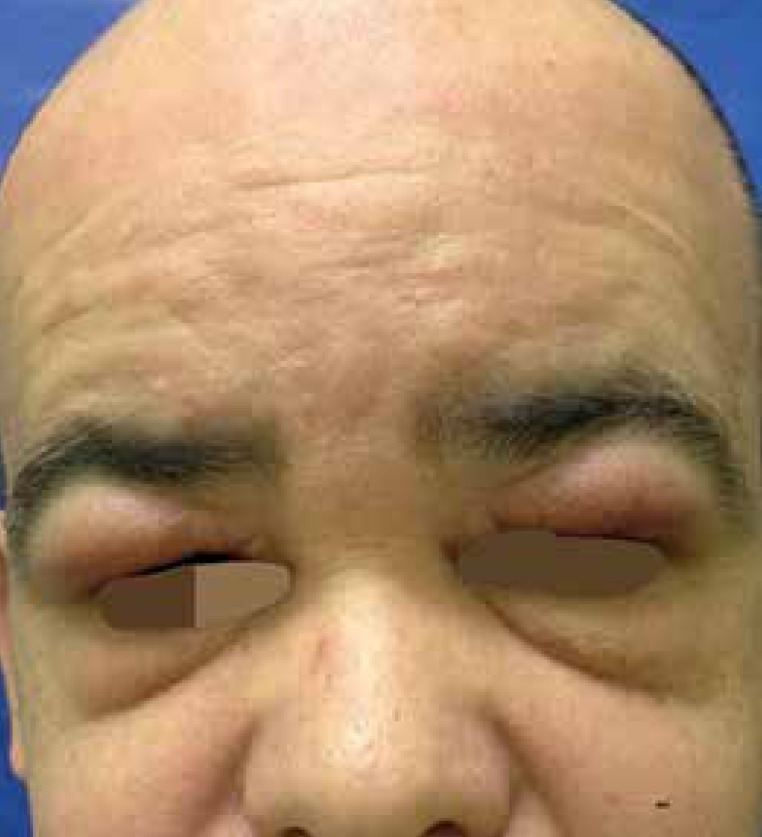
Erythema and edema on the upper eyelids

**Figure 2:**
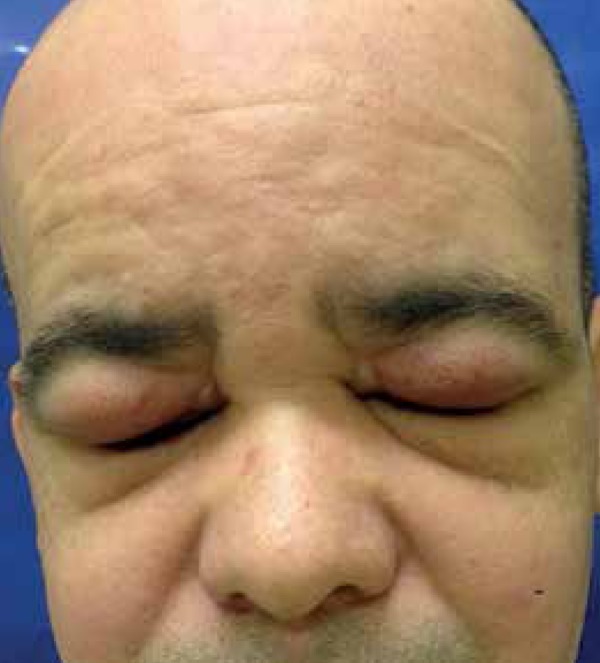
Erythema and edema on the upper eyelids Ectasia of the superficial vascular plexus

The patient stated that he had used tetracycline twice a day for 30 days, in addition to soap and sunscreen with no improvement. Dermatological examination revealed erythema and edema on the upper eyelids (Figures 1 and 2).

**Figure 3:**
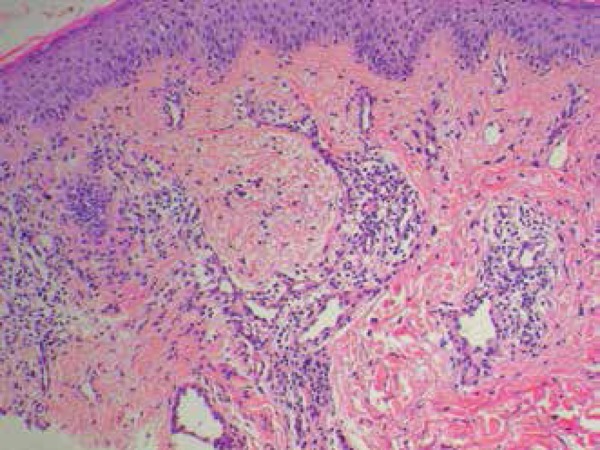
Tuberculoid focal granuloma

**Figure 4:**
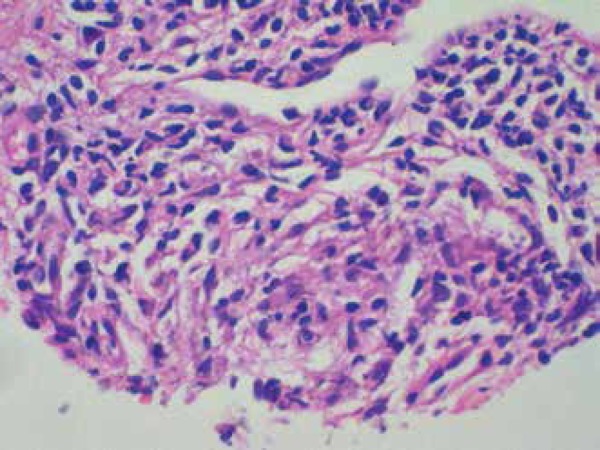
Mast cell staining with Giemsa

A biopsy showed a superficial dermatitis and perifolliculitis, focal granulomatous reaction, ectasia of the cutaneous superficial vascular plexus, and demodicosis corresponding histologically to a picture of rosacea (Figures 3 and 4).

**Figura 5:**
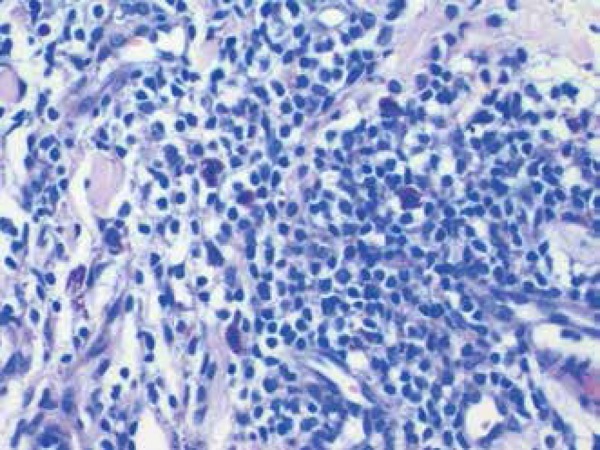
Mast cell staining with Giemsa

**Figure 6:**
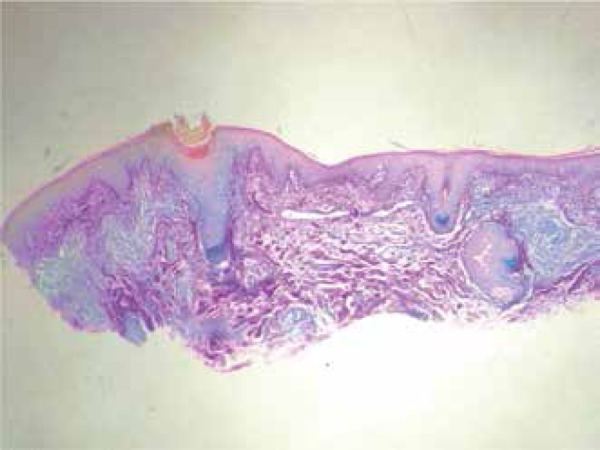
Staining with colloidal iron showing no increase in the amount of mucin in the dermis

Considering the hypothesis of Morbihan syndrome, we ordered new tests – including specific stains for mast cells and mucin, X-ray, thoracic CT, and biochemical tests – in order to rule out other diseases (Figures 5 and 6).^9^ All exams were within the normal range. The only change reported was the presence of mast cells in Giemsa staining, which, together with the pathological and clinical results, confirmed the diagnosis of Morbihan syndrome.

## DISCUSSION

Morbihan syndrome is characterized by the development of a hardened edema mainly on the upper half of the face. The disease usually occurs by the third or fourth decades of life and is more frequent in women.^10^ However, we reported a male patient with the same clinical features of the disease. It was initially thought to be rosacea. However, given the years of evolution with no improvement after antibiotic treatment, we considered the diagnosis of Morbihan syndrome and requested biopsy with suggestive results.

Pathological examination, although non-specific, is characterized by perivascular dermal edema with a lymphohistiocytic periannexal infiltrate containing numerous mast cells and dilation of lymphatic vessels. Granulomas are sometimes present, and sebaceous gland hyperplasia can be observed in patients who have had or have associated rosacea.^7^

Treatment, as confirmed by the literature, is challenging and the evidence base is very limited. The commonly adopted therapies include the control of the underlying inflammatory rosacea with broad-spectrum antibiotics and facial massage to improve drainage. Several systemic drugs have been used including thalidomide, clofazimine, tetracyclines, and steroids.^8^ However, only isotretinoin – alone or associated with ketotifen – has been reported to be effective at a dose ranging from 10-20 mg daily for 3-6 months in combination with ketotifen (1 mg twice daily) with little response though.^6^

The effectiveness of ketotifen may result from the direct interference with mast cell degranulation, which may be necessary for the collagen deposition and fibrotic reactions.^7^ Surgical treatments and CO2 laser have also been reported as treatment options, but information about success rates are not available yet.^8^
